# Interprofessional Health Education in the Region of the Americas

**DOI:** 10.1590/1518-8345.0000.3013

**Published:** 2018-05-07

**Authors:** Fernando Antonio Menezes da Silva, Silvia Helena De Bortoli Cassiani, José Rodrigues Freire

**Affiliations:** 1 PhD, Unit Chief, Unit of Human Resources for Health (HSS/HR), Department of Health Systems and Services (HSS), Pan American Health Organization/World Health Organization (PAHO/WHO), Washington, DC, United States of America. Email: menezesf@paho.org; 2 PhD, Regional Advisor on Nursing and Allied Health Personnel, Pan American Health Organization/ World Health Organization (PAHO/WHO), Washington, DC, United States of America. Email: cassianis@paho.org; 3 MSc, International Consultant, Unit of Human Resources for Health (HSS/HR), Department of Health Systems and Services (HSS), Pan American Health Organization/World Health Organization (PAHO/WHO), Washington, DC, United States of America. Email: rodrigujos@paho.org

**1:**
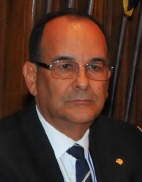

**2:**
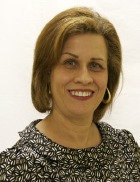

**3:**
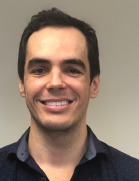

In the last two years, progress has been observed in the incorporation of Interprofessional Education (IPE) into policies on human resources for health in the countries of the Region of the Americas.

The Pan American Health Organization/ World Health Organization (PAHO/WHO) has encouraged its Member States to adopt the approach and support policymakers in expanding its use^1^. 

PAHO’s Strategy on Human Resources for Universal Access to Health and Universal Health Coverage^2^, recently adopted by Resolution CSP29.R.15, encourages countries to promote the development of interprofessional teams in service networks using IPE and diversified learning settings, with a focus on research, the sharing of experiences, and cooperation. 

PAHO/WHO has adopted a series of initiatives to provide policymakers with proposals to establish commitments to incorporate IPE as an innovative approach to the transformation of health systems, with the above-mentioned strategy as a point of reference. 

Among other actions, on 5-6 December 2017, the second regional technical meeting on IPE was held in Brasilia, Brazil. The event, organized jointly with the Ministry of Health of Brazil, was attended by representatives from different parts of the world, 22 of them from countries of the Region of the Americas. The purpose of the meeting was to discuss processes for incorporating IPE into policies on human resources for health, to establish a common agenda for strengthening IPE in the Region of the Americas, to foster the preparation of action plans to implement the approach, and to formalize the establishment of the Regional Network for Interprofessional Education in the Americas and the approval of its directives. 

As a result, 18 countries presented action plans for the implementation of IPE in their health policies between 2018 and 2019. The content of the proposals, for the most part, includes aspects that denote the clear commitment of governments to defining national policies that promote the adoption of IPE by educational and health institutions, promoting activities to strengthen institutional support, review curricular content, and develop teaching staff capable of working with IPE. 

According to the WHO 2010 Framework for Action on Interprofessional Education and Collaborative Practice(3), EIP can be difficult to explain, understand, and implement, since health professionals believe they are acting collaboratively, when in fact they simply work together with other professionals from a multiprofessional perspective^4^. However, what is seen in the Region of the Americas, after eight years of this publication, is that the theme is gaining visibility and reaching discussions at the political and academic levels. 

The challenge now is to give continuity to the plan, and results have already been achieved. The Regional Network for Interprofessional Education in the Americas (REIP)^5^, coordinated by Argentina, Brazil, and Chile, presented its application to become a member of the World Coordinating Committee “All Together Better Health” (WCC-ATBH), which consists of an organization comprised of regional networks focused on interprofessional practice and education in health, and includes representatives from around the world, which could greatly strengthen the exchange of experiences on IPE in the Region of the Americas.

Bolivia, Brazil, Cuba, Chile, Honduras, and Peru are moving forward with proposals to incorporate IPE into the curricular guidelines of undergraduate courses in the area of health and to formulate proposals for teacher qualifications. Argentina and Guiana are discussing proposals for carrying out research in this area. And Guatemala, Nicaragua, Panama, and Venezuela have presented strategies for the qualification of health service professionals, making use of the theoretical and methodological bases of IPE. 

Some countries, such as the Dominican Republic and Suriname, are proposing the establishment of National IPE Networks, while others, such as Paraguay, Uruguay, Colombia, and Costa Rica, are conducting surveys on the subject at the national level. 

It is expected that, though cooperation with PAHO/WHO, countries will be able to implement IPE as a potential approach to strengthening their health systems. In the current global context, it is no longer enough for health professionals to be more professional; they also need to be interprofessional. 
